# Apertures in the *Clostridium sporogenes* spore coat and exosporium align to facilitate emergence of the vegetative cell

**DOI:** 10.1016/j.fm.2015.04.013

**Published:** 2015-10

**Authors:** Jason Brunt, Kathryn L. Cross, Michael W. Peck

**Affiliations:** Institute of Food Research (IFR), Norwich Research Park, Colney, Norwich NR4 7UA, United Kingdom

**Keywords:** Spores, Exosporium, *Clostridium*, Coat, Germination, Sporiduct, Electron microscopy

## Abstract

*Clostridium sporogenes* forms highly heat resistant endospores, enabling this bacterium to survive adverse conditions. Subsequently, spores may germinate, giving rise to vegetative cells that multiply and lead to food spoilage. Electron microscopy was used to visualise changes in spore structures during germination, emergence and outgrowth. *C. sporogenes* spores were surrounded by an exosporium that was oval in shape and typically 3 μm in length. An aperture of 0.3–0.4 μm was observed at one end of the exosporium. The rupture of the spore coats occurs adjacent to the opening in the exosporium. The germinated cell emerges through this hole in the spore coat and then through the pre-existing aperture in the exosporium, before eventually being released, leaving behind a largely intact exosporium with an enlarged aperture (0.7–1.0 μm) and coat shell. The formation of this aperture, its function and its alignment with the spore coat is discussed.

## Highlights

• Electron microscopy was used to study structures of *Clostridium sporogenes* spores.
• Spore exosporia possessed either a terminal aperture or a lipped protrusion.
• Cells emerged through the preformed aperture or sporiduct of the spore.
• Apertures in the spore coat and exosporium were aligned, and may be predetermined.

## 1 Introduction

*Clostridium sporogenes* is a Gram positive anaerobic spore-forming bacterium. It is a significant cause of food spoilage (McClure, 2006), and occasionally pathogenic (Inkster et al., 2011), and because of its physiological and genetic similarity to *Clostridium botulinum* Group I (proteolytic *C. botulinum*) (Bradbury et al., 2012; Carter et al., 2009; Collins et al., 1994; Peck et al., 2011) it is often used as a surrogate for this organism in demonstrating the effectiveness of food processes (Brown et al., 2012; Diao et al., 2014; Taylor et al., 2013).

Clostridial spores persist in the environment. This highly resistant dormant state permits survival in hostile conditions (e.g. oxygen, absence of nutrients, heat treatment, desiccation, high pressure, toxic chemicals) that would be lethal to vegetative cells. The resistance properties of the spores of *C. sporogenes* and *C. botulinum* are a principal reason why these bacteria present a significant food spoilage and food safety problem (Brunt et al., 2014; Peck, 2009).

The ultrastructure of clostridial and bacillus spores is generally conserved (Henriques and Moran, 2007; Paredes-Sabja et al., 2014), and comprises a spore coat, outer membrane, cortex, inner membrane and spore core (Leggett et al., 2012). In contrast, the outermost layers, in particular the exosporium, vary for individual species and strains (Driks, 1999; Henriques and Moran, 2007; Paredes-Sabja et al., 2014). Indeed, *Bacillus subtilis* may not have a recognised exosporium (Driks, 1999), although a point of much debate (Henriques and Moran, 2007). The exosporium and coat constituents are synthesised in the mother cell and assembled around the developing forespore (Henriques and Moran, 2007). Electron microscopy has shown the exosporium in several clostridia to be loosely fitted around the spore with an opening at one of the polar regions (Lund et al., 1978; Mackey and Morris, 1972a,b; Stevenson and Vaughn, 1972). However, in *Clostridium difficile* the exosporium often lacks the gap that separates the spore coat from the exosporium which is observed in *Bacillus cereus* and *Bacillus anthracis* spores (Paredes-Sabja et al., 2014). Although the exact role of the exosporium is not completely understood, previous studies with *C. sporogenes* suggest that the exosporium may have a role in germination, outgrowth and attachment (Panessa-Warren et al., 1994, 1997). While the proteinaceous spore coat provides protection for the spore, the exosporium is the first point of contact of the spore with its environment. When conditions in the environment become favourable, the dormancy of bacterial spores is broken, germination occurs and cell multiplication recommences.

Despite the fact that extensive investigations have been undertaken on spore germination, coat structure, and clostridial cell growth from spores (Henriques and Moran, 2007; Setlow, 2014; Stringer et al., 2011; Webb et al., 2012), there is a relative lack of information regarding the cell's exit from its protective coats. The purpose of the present study was to build on previous observations in *C. sporogenes*, *Clostridium pasteurianum* and *B. anthracis* that the spore exosporium may have a hole or “bottle cap” at its pole (Mackey and Morris, 1972b; Panessa-Warren et al., 1994, 1997; Steichen et al., 2007). In particular we show that the rupture of the *C. sporogenes* spore coat occurs adjacent to the pre-formed aperture in the exosporium, and that the germinated cell emerges through aligned openings in the spore coat and the exosporium.

## 2 Materials and methods

### 2.1 Preparation of spores

*C. sporogenes* strain ATCC15579 was grown anaerobically at 37 °C in tryptone–yeast medium (TY). Spores of *C. sporogenes* were prepared in Robertson's cooked meat broth (Southern Group Laboratories) incubated at 37 °C for a period of 10 days. Spores, vegetative cells and debris, were harvested by filtration (20 μm) and cleaned using discontinuous density gradient centrifugation (Plowman and Peck, 2002). The pellets were washed six times in 50 ml chilled sterile water, with centrifugation at 2000 × *g* (10 min). After each centrifugation step the top layer of the pellet, consisting predominantly of vegetative cells, was discarded and the bottom layer resuspended. Pellets were finally resuspended in 2 ml water, layered onto 10 ml 50% (v/v) aqueous solution of sodium/meglumine diatrizoate (Urografin 370, Schering, Germany) and centrifuged (6000 × *g*, 4 °C, 40 min). The top layers containing debris, vegetative cells and germinated spores were removed, while the pellet, consisting predominately of phase-bright spores, was resuspended in 50 ml water. Pellets were then washed a further six times in sterile water (2000 × *g*, 4 °C, 10 min), resuspended in 2 ml water and stored at 2 °C. Microscopic examination confirmed that the suspensions consisted of >99% phase-bright spores. Two distinct crops of spores were tested and gave identical results. Prior to use, spore suspensions were enumerated using a haemocytometer and adjusted to a final concentration of ∼1 × 10^8^ spores/ml.

### 2.2 Spore germination

Samples to visualise only germination were prepared for transmission electron microscopy (TEM) and scanning electron microscopy (SEM). Spores were heat activated (80 °C, 15 min), and incubated (12 h) aerobically at 30 °C in a filter sterilised (0.45-μm syringe filter, Millipore) germinant solution comprising l-alanine (100 mM), l-lactate (50 mM) and NaHCO_3_ (50 mM) in Tris–HCl buffer (20 mM, pH 7.4) (Sigma). Samples (500 μl) were then removed and prepared for TEM and SEM. To prepare samples to visualise germination and subsequent emergence and outgrowth, SEM was used. Spore suspensions were heat activated (80 °C, 15 min), and incubated anaerobically at 30 °C in TY broth plus l-alanine (100 mM) and l-lactate (50 mM) to observe emergence and outgrowth. Samples (500 μl) were taken at 30 min intervals for 5 h, centrifuged (4000 × *g*, 2 min, 4 °C), and the remaining pellet prepared for SEM.

### 2.3 Transmission and scanning electron microscopy

The bacterial spore suspensions were fixed in 2.5% glutaraldehyde in 0.1 M PIPES (pH 7.4) buffer in 1.5 ml polypropylene tubes for a minimum of 2 h. The suspensions were centrifuged (5400 × *g*, 2 min) and resuspended in 0.1 M PIPES (pH 7.4) buffer three times. After the final centrifugation, the buffer was removed to leave a pellet.

For TEM, the cell pellets were mixed 1:1 with molten 2% low gelling temperature agarose (Type VII, A-4018, Sigma), which was solidified by chilling, then chopped into small pieces (approximately 1 mm^3^). The sample pieces were post-fixed in 1% aqueous osmium tetroxide for 2 h then, after buffer washes, dehydrated through a series of ethanol solutions (10, 20, 30, 40, 50, 60, 70, 80, 90, 3 × 100%). After the third change of 100% ethanol, the ethanol was replaced with a 1:1 mix of 100% ethanol to LR White medium grade resin and put on a rotator for 1 h. This was followed by a 1:2 and a 1:3 mix of 100% ethanol to LR White resin and finally 100% resin, with at least 1 h between each change. The resin was changed twice more with fresh 100% resin with periods of at least 8 h between changes. The sample pieces were each transferred into BEEM embedding capsules with fresh resin and polymerised overnight at 60 °C. Sections approximately 90 nm thick were cut using an ultra-microtome (Ultracut E, Reichert-Jung) with a glass knife, collected on formvar/carbon coated copper grids, and stained sequentially with uranyl acetate and lead citrate. The sections were examined and imaged in a FEI Tecnai G2 20 Twin transmission electron microscope at 200 kV.

For SEM, each cell pellet was transferred onto the centre of a small square of filter paper which had been folded into three equal parts in both directions and then opened out. The filter paper was then folded and inserted into a critical point drying capsule and dehydrated in a series of ethanol solutions (10, 20, 30, 40, 50, 60, 70, 80, 90, 3 × 100%). Samples were dried in a Leica EM CPD300 Critical Point Dryer using liquid carbon dioxide as the transition fluid. The parcels were carefully unfolded in a clean plastic Petri dish and the dry cells were mounted onto SEM stubs via sticky tabs by flicking the back of the filter paper in the direction of the stub. The samples were coated with gold in an Agar high resolution sputter-coater apparatus. Scanning electron microscopy was carried out using a Zeiss Supra 55 VP FEG SEM, operating at 3 kV.

## 3 Results/discussion

A key feature that has contributed to the success of clostridia is their ability to form highly resistant endospores. Under suitable conditions the spores germinate with associated loss of resistance properties, and cell multiplication recommences. To understand more about these processes, transmission and scanning electron microscopy were used to visualise structural changes during germination, emergence and outgrowth.

**Fig. 1:**
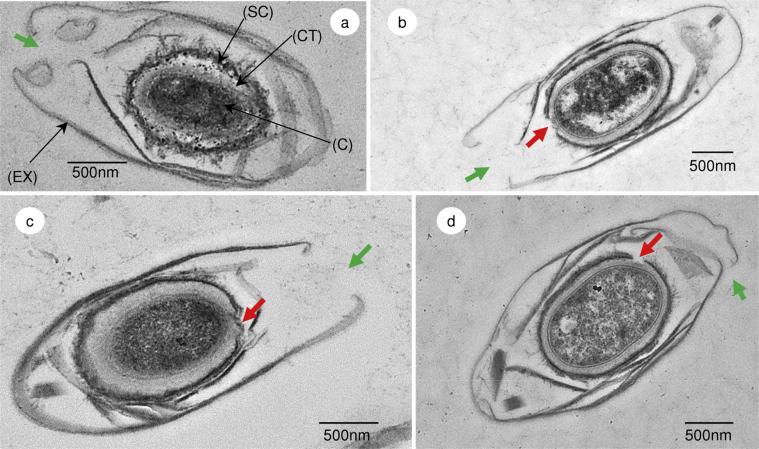
Transmission electron micrograph images of *C. sporogenes* spores. (a) Transmission electron micrograph of a cross section of a *C. sporogenes* spore with the cortex intact. (CT) cortex; (SC) spore coat; (C) spore core; (EX) exosporium. (b), (c) and (d) TEM of germinating *C. sporogenes* spores (l-alanine (100 mM)/l-lactate (50 mM)/NaHCO_3_ (50 mM), 20 mM Tris buffer, pH 7.4) showing expansion of the spore cortex and spore core. Red arrow indicates breaks and distortion of the spore coat. Green arrow indicates open section in the exosporium which may allow the release of the newly formed cell.

Transmission electron microscopy (TEM) was utilized to visualise the changes in the spore structures of *C. sporogenes* during germination (Fig. 1). The TEM of the dormant spore showed the exosporium, cortex, outer membrane and spore core (Fig. 1a). Comparison of the germinating spore with a dormant spore highlighted the enlarged, less dense spore core, signifying its hydration (Fig. 1b,c,d). This morphological arrangement appears to be conserved in *C. pasteurianum*, *C. botulinum*, *C. sporogenes* and *Clostridium novyi* (Mackey and Morris, 1972a; Montville et al., 1985; Panessa-Warren et al., 1994; Plomp et al., 2007). Moreover, the exosporium appearance observed here differs from that of *C. difficile* which is often described as less organised and lacking a clear gap/inner coat space observed in other species (Paredes-Sabja et al., 2014). Interestingly, there was a disruption in the multi-layered coat of the germinating spore, and an aperture in the exosporium that aligned with the break in the spore coat (Fig. 1b,c,d). Of the small number of spores that were in the correct orientation, so as to visualise the exosporium aperture, all showed a rupture in their coats adjacent to the aperture. In comparison, Mackey and Morris (1972a) observed numerous breaks in the spore coat of *C. pasteurianum* before the cell emerged through the open base of the exosporium. An opening in the exosporium, visualised by TEM, has also been reported in strains of *C. pasteurianum* (Mackey and Morris, 1972a) *C. botulinum* Group I (Stevenson and Vaughn, 1972), and *C. botulinum* Group I and *C. botulinum* Group II (S. C. Stringer, M. D. Webb, unpublished data).

**Fig. 2:**
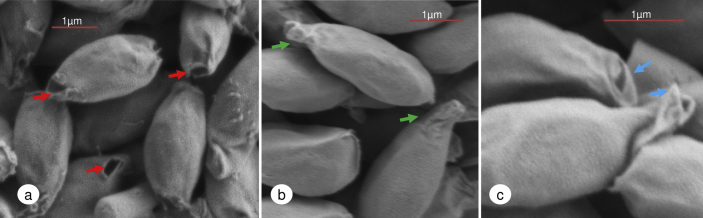
Scanning electron microscopy of *C. sporogenes* dormant spores surrounded by their exosporium. (a) Red arrows point to a small aperture (0.3–0.4 μm) at one end of the exosporium. (b) Projections at one end of the exosporium. Green arrows point to a lipped terminal protrusion (sporiduct). (c) Magnified image of the lipped terminal protrusion. Blue arrows indicate a lipped end with a small opening.

Scanning electron microscopy (SEM) was used to investigate further the structure and function of the aperture (Fig. 2). *C. sporogenes* spores were surrounded by an exosporium that was oval in shape and typically up to 3 μm in length. The exosporium covers the entire spore and has a rough textured appearance. Several species of *Clostridium* have an exosporium including *C. botulinum* Groups I and II (Lund and Peck, 2000; Montville et al., 1985; Stevenson and Vaughn, 1972; Takumi et al., 1979), *C. sporogenes* (Panessa-Warren et al., 1994, 1997), *C. pasteurianum* (Mackey and Morris, 1972a,b), *C. novyi* (Plomp et al., 2007) and *C. difficile* (Panessa-Warren et al., 1997). The function of the exosporium is poorly understood, and is reported to provide a barrier to large molecules (e.g. antibodies, enzymes) (Steichen et al., 2005) but not smaller spore germinants, and play a role in spore adhesion (Faille et al., 2007; Lequette et al., 2011). With regards to this study, the aperture observed in the exosporium would almost certainly allow entry of larger molecules into the exosporium and therefore its role as a protective barrier, at least with regards to this strain, is limited. A number of proteins have been identified in the exosporium that have been implicated in spore germination (Terry et al., 2011). Following germination the developing cell was previously reported to exit the exosporium prior to elongation and cell division (Steichen et al., 2007).

Our images revealed that ungerminated *C. sporogenes* spores had one of two terminal structures at one end of the exosporium; either an aperture (Fig. 2a) of 0.3–0.4 μm at its widest point or a lipped terminal protrusion termed here a sporiduct (Fig. 2b and c). The fraction of *C. sporogenes* spores exhibiting a hole or sporiduct in the exosporium could not be reliably estimated due to spore orientation. Although the significance of the spore having a sporiduct as opposed to an aperture is unknown, we can only hypothesize that the sporiduct could be a relatively weak structure and removed during spore preparation to leave an aperture. The sporiduct structure itself had a loose or flaccid appearance with a lipped protrusion and opening (Fig. 2c). Some researchers have speculated that a similar “tail” region observed in some clostridia spores, although different in morphology, may allow the release of the newly formed cell (Mackey and Morris, 1972a; Panessa-Warren et al., 1994). Furthermore, a “bottlecap” at the end of spores of *B*. *anthracis* has been proposed (Steichen et al., 2007). This aperture and sporiduct were observed in dormant (ungerminated) spores as well as in germinating spores of *C. sporogenes*, although previously Panessa-Warren et al. (1994) observed that the dormant spores were surrounded by a continuous exosporium with a blunt tail region, with the exosporial opening only visible once germination had commenced. The difference between these two studies may reflect strain variability.

**Fig. 3:**
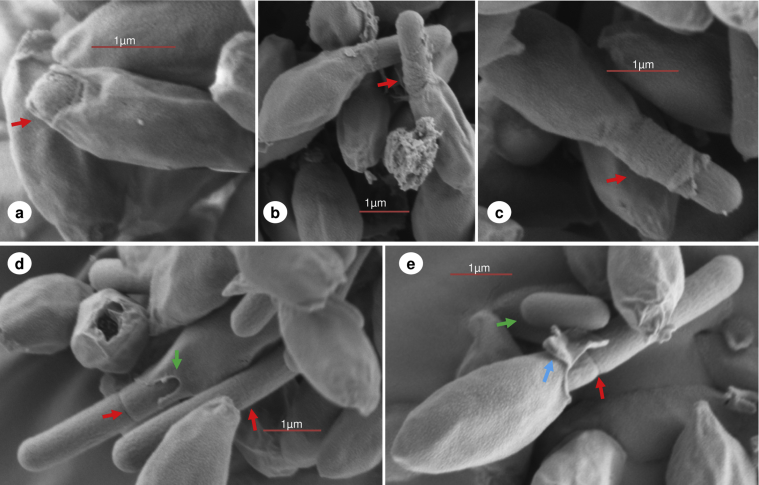
Scanning electron microscopy of *C. sporogenes* germinated (anaerobic TY broth plus l-alanine (100 mM) and l-lactate (50 mM)) spores and outgrowing cells. (a) emergence of the newly formed cell (red arrow) with enlargement of the aperture. (b) Continued outgrowth of the cell with detritus material attached (red arrow) to the outer regions of the cell. (c) Elongation of the cell with distending sporiduct (red arrow). (d) Cells showing septum formation (red arrows) and tearing of the exosporium aperture (green arrow). (e) Continued elongation of the cell showing septum formation (red arrow) while still partially interned in the exosporium case. The sporiduct has enlarged and torn (blue arrow). Free cells are also evident in samples (green arrow).

**Fig. 4:**
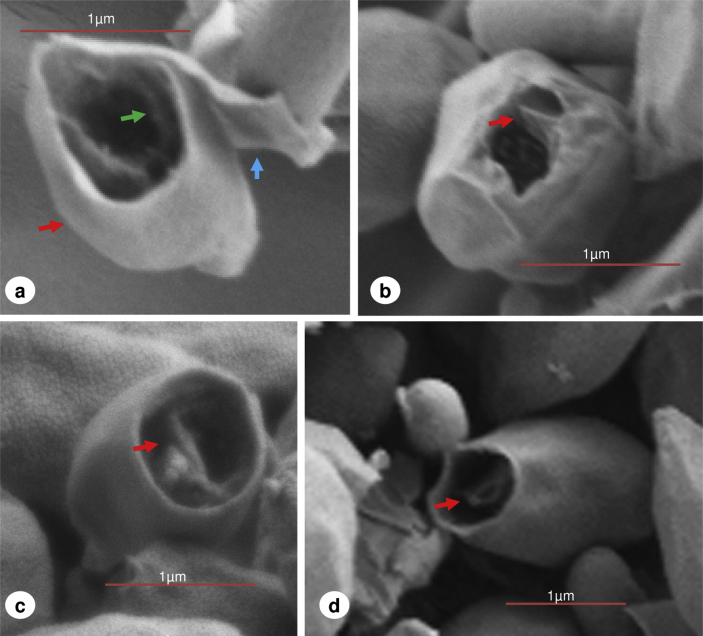
Scanning electron microscopy of *C. sporogenes* vacated exosporium casings and augmented sporiduct. (a) Inside the exosporium casing (red arrow) an open tubular structure (green arrow) is observed which is partially detached from the exosporium. Blue arrow indicates possible torn remnants of the sporiduct. Images b, c and d are the various structures observed (red arrows) inside the exosporium casings.

Having ascertained that ungerminated spores of *C. sporogenes* possess an aperture or sporiduct at the terminal of the exosporium, we tested the hypothesis that the newly formed cell would emerge through this structure. *C. sporogenes* spores were incubated in anaerobic TY medium plus l-alanine and l-lactate which had previously been shown to be effective conditions for germination and outgrowth of *C. sporogenes* spores (Brunt et al., 2014). Samples were taken periodically for scanning electron microscopy. However, emergence and outgrowth were not synchronous and samples contained spores and cells at various stages of morphological development. Emergence of the newly formed cell occurred through the preformed aperture or sporiduct of the spore (Fig. 3) forcing the aperture to distend and the sporiduct to stretch (Fig. 3a and c). As the cell continued to elongate, emerging cells had remnants of presumably the spore cell wall or cortical elements attached (Fig. 3b). In general the cells continued to grow and elongate from within the exosporium case (Fig. 3d and e). Moreover, a large proportion of these emerging cells were already showing septum formation (Fig. 3d and e). A comparable study with *B. anthracis* had previously shown that the outgrowing expanding single cell bursts from its coat and exosporium case through a preformed cap in the exosporium before elongation and division (Steichen et al., 2005, 2007). Similarly, *C. botulinum* Group II cells also appear to burst out of their coat and exosporium, before elongation and division (S. C. Stringer, M. D. Webb, unpublished data). Furthermore, previous observations with *C. sporogenes* concluded that emergence of the cell was due to proteases that digested the spore coats and then the exosporium (Hoeniger and Headley, 1969). In comparison our findings show that, at least in this strain, *C. sporogenes* emergence is a slow process of sufficient duration to allow the cell to form a septum before eventual release from the exosporium. We have also observed similar gradual cell release from the exosporium, by phase contrast microscopy, with *B. cereus* (S. C. Stringer, M. D. Webb, unpublished data). Emerging cells always exited through the pre-existing aperture or sporiduct leaving behind an exosporium shell (Fig. 4). These “empty” structures were only observed in the 5hr sample time points at which stage a large proportion of free cells were evident. Furthermore, the aperture had in increased in size (0.7–1.0 μm, at its widest point) perhaps due to the widening during cell emergence. Inside the exosporium shell various structures were perceived (Fig. 4a, b, c & d). The inner open tubular structures that appear to be at least partially detached from the exosporium may represent remnant spore coats which were left behind following germination and emergence. TEM studies have shown that a considerable portion of the spore coat remained within the exosporium following cell emergence of *C. pasteurianum* ATCC 6013 (Mackey and Morris, 1972a), *C. botulinum* Group I ATCC 3502 and *C. botulinum* Group II Eklund17B (S. C. Stringer, M. D. Webb, unpublished data). Interestingly, as the hole of the tubular spore coat structure is aligned with the opening in the exosporium structure, this alignment may be genetically predetermined.

The findings presented here establish the role of the exosporium and spore coats in germination and cell emergence in *C. sporogenes*. Evidence is presented that the rupture of the spore coats occurs adjacent to the opening in the exosporium. It is hypothesised that this polarity is genetically pre-determined in the dormant spore. The cell emerges through this hole in the spore coat, then through the pre-existing aperture in the exosporium. Interestingly, the cell does not always exit immediately, with septated cells observed emerging through the exosporium. These findings lead to further important questions. For example if the hypothesis that the aperture/sporiduct is a deliberate preformed system designed to release the cell is correct, then which proteins are responsible for forming this structure? At what point is the structure assembled within the mother cell? To establish the role of the exosporium and spore coats in germination and cell emergence in *C. sporogenes* more in depth studies are required to understand the mechanisms by which vegetative cells are released from the *C. sporogenes* exosporium structure. Furthermore, more evidence is needed to confirm that all germinated spores release vegetative cells through their sporiduct. What is clear is that the structure and function of the exosporium deserves more detailed scientific investigation.
